# *Porphyromonas gingivalis* traffics into endoplasmic reticulum-rich-autophagosomes for successful survival in human gingival epithelial cells

**DOI:** 10.1080/21505594.2018.1454171

**Published:** 2018-04-04

**Authors:** Kyulim Lee, JoAnn S. Roberts, Chul Hee Choi, Kalina R. Atanasova, Özlem Yilmaz

**Affiliations:** a Department of Oral Biology, University of Florida, Gainesville, Florida, USA; b Department of Oral Health Sciences, Medical University of South Carolina, Charleston, South Carolina, USA; c Department of Microbiology and Medical Science, Chungnam National University, School of Medicine, Daejeon, Republic of Korea; d Department of Periodontology, University of Florida, Gainesville, Florida, USA; e Microbiology and Immunology, Medical University of South Carolina, South Carolina, USA

**Keywords:** *Porphyromonas gingivalis*, opportunistic bacteria, intracellular survival, autophagy, epithelial cells, oral mucosa, persistence

## Abstract

*Porphyromonas gingivalis*, an opportunistic pathogen usurps gingival epithelial cells (GECs) as primary intracellular niche for its colonization in the oral mucosa. However, the precise characterization of the intracellular trafficking and fate of *P. gingivalis* in GECs remains incomplete. Therefore, we employed high-resolution three-dimensional-transmission-electron-microscopy to determine the subcellular location of *P. gingivalis* in human primary GECs upon invasion. Serial sections of infected-GECs and their tomographic reconstruction depicted ER-rich-double-membrane autophagosomal-vacuoles harboring *P. gingivalis*. Western-blotting and fluorescence confocal microscopy showed that *P. gingivalis* significantly induces LC3-lipidation in a time-dependent-manner and co-localizes with LC3, ER-lumen-protein Bip, or ER-tracker, which are major components of the phagophore membrane. Furthermore, GECs that were infected with FMN-green-fluorescent transformant-strain (PgFbFP) and selectively permeabilized by digitonin showed rapidly increasing large numbers of double-membrane-vacuolar-*P. gingivalis* over 24 hours of infection with a low-ratio of cytosolically free-bacteria. Moreover, inhibition of autophagy using 3-methyladenine or ATG5 siRNA significantly reduced the viability of intracellular *P. gingivalis* in GECs as determined by an antibiotic-protection-assay. Lysosomal marker, LAMP-1, showed a low-degree colocalization with *P. gingivalis* (∼20%). PgFbFP was used to investigate the fate of vacuolar- versus cytosolic-*P. gingivalis* by their association with ubiquitin-binding-adaptor-proteins, NDP52 and p62. Only cytosolic-*P. gingivalis* had a significant association with both markers, which suggests cytosolically-free bacteria are likely destined to the lysosomal-degradation pathway whereas the vacuolar-*P. gingivalis* survives. Therefore, the results reveal a novel mechanism for *P. gingivalis* survival in GECs by harnessing host autophagy machinery to establish a successful replicative niche and persistence in the oral mucosa.

## Introduction

*Porphyromonas gingivalis* is a Gram (-) facultatively intracellular opportunistic pathogen linked to several systemic chronic diseases such as rheumatoid arthritis, diabetes, and cancer [1]. It is a proposed keystone pathogen due to its demonstrated ability to promote a favorable host microbial environment conducive for disease [2,3]. In the oral mucosa, epithelial cells have been considered to be a major intracellular niche for *P. gingivalis* [4-7] where the microorganism has been shown to successfully invade, replicate, and survive in human primary gingival epithelial cells (GECs), and later can spread intercellularly through actin formed structures [8]. Initial internalization of *P. gingivalis* into GECs is very rapid and requires both the host and bacteria to be metabolically active [9]. Furthermore, *P. gingivalis* invasion into GECs is not associated with the endocytic pathway and primarily requires specific binding to Beta-1 integrins on the cell surface through *P. gingivalis* fimbriae [10–13]. In addition, the inhibition of cytoskeletal rearrangements significantly impedes *P. gingivalis*' ability to invade primary GECs [9,10,14].

Once invaded, this opportunistic pathogen can manipulate the host machinery to facilitate its long-term survival by inhibiting the intrinsic apoptotic pathway through reduced cytochrome *c* release and caspase 3/9 activation [15–17]; modulating extracellular ATP-induced cellular reactive oxygen species and oxidative stress pathways [18–25]; and attenuating activation of NLPR3 inflammasome pathways thereby reducing pro-inflammatory cytokine IL-1β secretion [19,21–23,26]. Moreover, *P. gingivalis* promotes survival and proliferation of primary gingival epithelial cells through activation of the Phosphatidylinositol-4,5-bisphosphate 3-kinase (PI3K)/protein-kinase B (Akt) pathway [15,16,27] which prevents the activity of pro-apoptotic Bad and promotes upregulation of cell cycle proliferative components [28,29]. Most recently, intracellular *P. gingivalis* has been shown to inhibit NOX2-Reactive-Oxygen-Species (ROS) and subsequent hypochlorous acid production, thereby evading host bacterial elimination [24]. Therefore, *P. gingivalis* has multiple strategies by which it avoids immune surveillance and can successfully survive in primary GECs.

Epithelial cells are now recognized as a major arm of innate immunity whereas these cells can be usurped by opportunistic pathogens to provide a safe haven for bacterial persistence in the mucosal tissues. Similarly, *P. gingivalis* can evade host-mediated defense systems through its secreted effectors, converting epithelial cells into a prime reservoir for the organism's survival and further propagation in the oral mucosa [7,17,20–22,24,27,28,30,31]. Although the molecular cell biology of *P. gingivalis* infection in human GECs has been largely characterized, especially for establishing long term survival, only little attention has been given to the intracellular trafficking and fate of the organism in these critically important host cells. *P. gingivalis* trafficking has been observed in cell types such as KB cells (HeLa contaminants [32]), dendritic cells, macrophages and endothelial cells, which reveals evidence suggestive of differential intracellular trafficking of *P. gingivalis* dependent on host-cell type [10,33–35].

We therefore study the intracellular trafficking and fate of *P. gingivalis* in human primary GECs. Bacteria have evolved many mechanisms to colonize host cells and live intracellularly. While the autophagy pathway is an intracellular degradation process playing a critical housekeeping role in maintaining cellular homeostasis, several chronic opportunistic pathogens have been reported to evade autophagic-recognition or utilize the autophagy machinery for their own survival, successfully replicating inside autophagosome-vacuoles [36]. The role of autophagy in bacterial pathogenesis appears to be multi-dimensional, shown by the characterization of several different autophagy pathways triggered by bacterial invasion leading to either bacterial degradation or enhanced bacterial survival [37]. *P. gingivalis* was initially hypothesized to be largely free in the cytosol throughout the infection in primary GECs [10]. However, in the present study we for the first time demonstrate that infection by *P. gingivalis* induces autophagy in GECs in a time-dependent manner. Our examinations reveal, through 3-dimensional morphological TEM and confocal analyses, the marked presence of *P. gingivalis* in ER-rich/LC3-positive autophagic vacuoles, the predominant subcellular location for the bacteria to survive in primary GECs. The amount of viable *P. gingivalis* was significantly decreased upon specific pharmacological or RNAi inhibitions of autophagy (using 3-methyladenine (3-MA) or siRNA targeting ATG5 respectively). Confocal analysis displayed less than 25% of the bacteria were associated with lysosomes at 24 hours. Only a small percentage (∼20%) of bacteria were detected free in the cytosol, also distinctly marked by the anti-microbial NDP52 and p62 ubiquitin-binding-adaptor proteins.

Thus, our findings reveal a novel mechanism for *P. gingivalis* successful intracellular survival in gingival epithelial cells and illustrate the protective ability of these autophagic replicative niches in shielding *P. gingivalis* from selective ubiquitin-mediated targeting for lysosomal degradation. Overall, this study provides new molecular insight into understanding the intracellular life and fate of *P. gingivalis* in the oral mucosa and offers an important framework for future mechanistic studies. This new knowledge may also lead to highly targeted therapeutic interventions for controlling opportunistic bacterial colonization in the mucosal cells by using specific autophagy inhibitors.

## Results

### P. *gingivalis* primarily traffics to double-membrane vacuoles over time of infection

**Figure 1.**
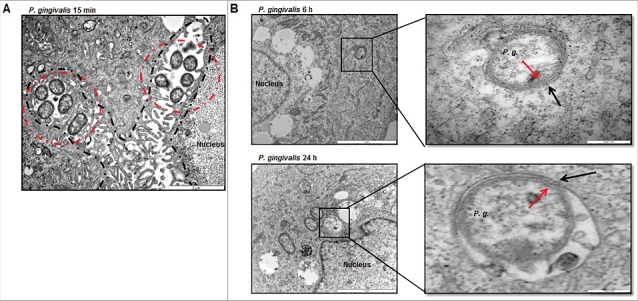
TEM analysis shows intracellular *P. gingivalis* localizes to ER-perinuclear regions and is encapsulated by double-membrane vacuoles. (A) Transmission Electron Microscopy (TEM) analysis indicates internalized bacteria being closely positioned in the ER-rich regions at 15 minutes post *P. gingivalis* infection (MOI 100). ER structure(s) are outlined in a dotted black line while intracellular bacteria are identified by dotted red circles. (B) Ultrastructural analysis by thin-section electron microscopy show majority of *P. gingivalis* being enclosed by double membranous structures (characteristic of autophagosomes) at 6 hour post-infection. Primary GECs were infected with *P. gingivalis* at MOI 100 for 6 hours (top) and 24 hours (bottom). Boxed areas show an enlarged region. Black arrows indicate the double membrane surrounding the bacteria and red arrows indicate the *P. gingivalis* membrane.

**Figure 2.**
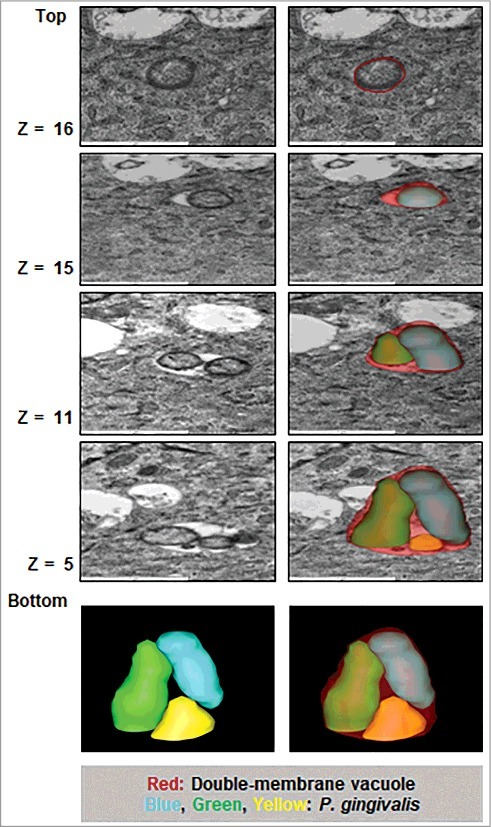
Three-dimensional (3D) TEM analysis shows double membrane-vacuoles in primary GECs harboring *P. gingivalis* contain multiple bacteria. At least 16 consecutive TEM sections were collected from cell samples infected with *P. gingivalis* for 12 hours at MOI 100 and micrographs of intracellular bacterial cells were captured. The 12 hour images were illustrated to highlight the presence of double-membrane replicative niches, appearing earlier than 24 hours post-infection (not shown) which also revealed similar results. Representative slices at stacks 5, 11, 15, and 16 are shown and the bacteria and phagosome are outlined using 3dmod software.

**Figure 3.**
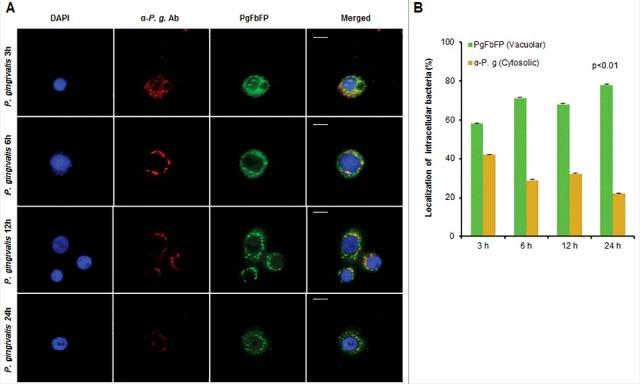
*P. gingivalis* vacuolar localization significantly increases over time of infection in primary GECs. Immunofluorescence intensity based quantification of cytoplasmic and vacuolar *P. gingivalis* at 3, 6, 12, and 24 hours post infection. (A) Primary GECs were infected with PgFbFP, green fluorescing *P. gingivalis* at MOI 100. Infected PgFbFP were labeled using anti-*P. gingivalis* antibody followed by Alexa 594 (red-fluorescence) secondary antibody after selective digitonin permeabilization (which will only permeabilize cellular plasma membrane). Cytoplasmic bacteria were detected as FbFP-and Alexa 594 positive (yellow), whereas vacuolar bacteria were solely FbFP-positive (green). 40x micrographs; Bar 10 µm. (B) The percentage of intracellular bacteria are represented as mean +/− SD; n > 3. *p* < 0.01 as determined by ANOVA. The slight decrease and increase in vacuolar and cytoplasmic bacteria, respectively, observed at 12 hours post-infection was not statistically significant compared to 6 hours post-infection (according to Student's two-tailed t-test).

Using Transmission Electron Microscopy (TEM) to determine the ultrastructural structures and subcellular localization of *P. gingivalis* in GECs, intracellular *P. gingivalis* was found to be localized in the perinuclear region of the host epithelial cell as early as 15 minutes post-infection. This finding was concordant with a previous report studying *P. gingivalis* invasion into human primary GECs through a real-time wide-field deconvolution microscopy [9]. The structures that localize and begin to envelope the bacteria appeared as endoplasmic reticula (ER), which are sites of initial phagophore formation and an important contributor to phagophore membrane composition [38,39] (Fig. 1A). At later times of infection, 6 and 24 hours, *P. gingivalis* is observed in double-membrane vacuoles resembling autophagosomes (Fig. 1B). Further examination of these autophagic vacuoles harboring *P. gingivalis* using 3-dimensional (3D) TEM, revealed multiple bacteria are present in the same vacuole (Fig. 2). Interestingly, prior to the 3D reconstruction of the same vacuole displaying multiple bacteria in it, the image taken from the top layers of the z-stack displayed merely a single *P. gingivalis* appeared as freely in the cytoplasm of the host cell. These ultrastructural data indicate that intracellular *P. gingivalis* can reside in double-membrane ER-rich autophagic vacuoles which may serve as a replicative niche for the opportunistic organism. We further identify, using quantitative immunofluorescence, whether *P. gingivalis* localizes predominantly inside double-membrane vacuoles or freely in the cytosol. We employed a novel tool established in our laboratory using a Flavin mononucleotide (FMN)-green-fluorescent transformant-strain of *P. gingivalis* (PgFbFP) [40] to both visualize and quantify metabolically active *P. gingivalis* in combination with selective permeabilization of the plasma membrane by digitonin [41]. Immunofluorescent staining with an anti- *P. gingivalis* antibody (red fluorescence) in the presence of digitonin ensures that only cytosolic bacteria are stained by the antibody (shown by yellow fluorescence) while the double-membrane vacuolar bacteria will remain unstained by the antibody and will intrinsically fluoresce green only since the vacuolar membranes remain intact. The results reveal that intracellular *P. gingivalis* resides predominantly inside these vacuoles, while only a small percentage of bacteria are freely found in the cytosol. Specifically, ∼60% of the metabolically active *P. gingivalis* resided in vacuoles at 3 hours of infection which then increased to ∼80% at 24 hours. This demonstrates that the localization of *P. gingivalis* in the double-membrane vacuoles increases significantly over time with a corresponding decrease in the number of cytosolic bacteria (Fig. 3).

### P.*gingivalis* co-localizes with autophagic and ER markers in primary GECs

**Figure 4.**
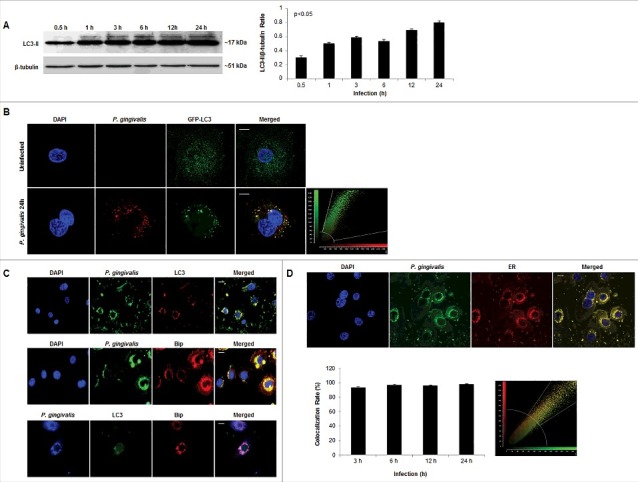
*P. gingivalis* significantly co-localizes with LC3 positive/ER regions in primary GECs. (A) Western blot analysis of LC3II lipidation of *P. gingivalis*-infected cells (MOI 100) over 0.5, 1, 3, 6, 12, and 24 hours of infection. Quantification was conducted using NIH ImageJ analysis; n≥3; p<0.05 as determined by ANOVA. (B) GFP-LC3 transfected GECs were infected with *P. gingivalis* 24 hours stained red (Alexa 594) and analyzed using confocal microscopy. Colocalization rate is ∼99%. 40x micrographs; Bar 10 µm. (C) *P. gingivalis* infected GECs, after 24 hours, were stained for LC3 (Alexa 594 or 488), ER lumen protein Bip (Alexa 594), and *P. gingivalis* (Alexa 488 or 350). High co-localization is observed through immunofluorescence imaging between *P. gingivalis* with LC3 and LC3 with Bip. 20x micrographs; Bar 10 µm. (D) Representative micrographs of 24 hour *P. gingivalis* infected cells (Alexa 488) and ER Tracker (red) analyzed through quantitative confocal microscopy. Quantitative analysis of 3, 6, 12, and 24 hours of infection demonstrate high, steady co-localization rate between *P. gingivalis* and the ER (co-localization ∼98%). 20x micrographs; Bar 10 µm.

These initial morphological and phenotypic characterizations, suggestive of ER-rich double-membrane autophagic vacuoles, incited our investigation into the specific identity of the vacuoles harboring *P. gingivalis* through analysis of the autophagic protein marker LC3, ER, and ER luminal Binding immunoglobulin protein (Bip). Western blot analysis revealed the significant (p<0.05) and steady increase in LC3II lipidation over the course of *P. gingivalis* infection in primary GECs (Fig. 4A). Overexpression of LC3 in primary GECs through transfection of Green-Fluorescent-Protein (GFP)-LC3 also showed a high colocalization rate with *P. gingivalis* (∼99%) at 24 hours infection (Fig. 4B) which is was also supported by immunofluorescence staining of endogenous LC3 (Fig. 4C). Volocity 3-dimensional analysis (PerkinElmer) of immunofluorescent images provided further evidence showing a high degree of *P. gingivalis* localization with LC3 puncta (Supplementary Figure 1). Consistent with the ultrastructural analysis showing *P. gingivalis* interacting with ER-like structures, *P. gingivalis* co-localizes with Bip, an ER lumen protein, which also displays a high degree of colocalization with LC3 (Fig. 4C). Further time kinetics analyses of *P. gingivalis* association with ER structures were analyzed through quantitative confocal microscopy by probing *P. gingivalis*-infected GECs with an ER-specific fluorescent dye, ER Tracker. Consistent with our previous findings [40], the confocal analysis displayed a steady level of high-degree co-localization (∼90%) between *P. gingivalis* and the ER over the 24 hour infection period (Fig. 4D). Taken together, these data support that the double-membrane vacuoles harboring *P. gingivalis* in primary GECs are autophagosomes formed using ER-membranes after infection.

### Inhibition of autophagic flux significantly diminishes *P. gingivalis* intracellular survival in primary GECs

**Figure 5.**
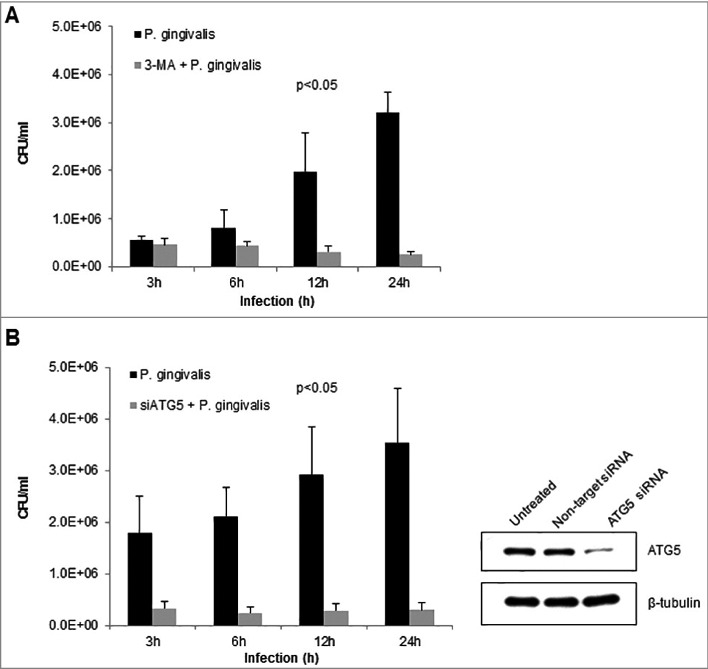
Inhibition of autophagy significantly decreases intracellular survival of *P. gingivalis* in primary GECs. Primary GECs were infected with *P. gingivalis* at an MOI 100 for 3, 6, 12, and 24 hours. *P. gingivalis* survival was determined using a standard antibiotic protection assay and quantified by colony forming units (CFU)/ml based on blood agar plate counts. Graphs are represented as mean +/ SEM, n≥3; *p* < 0.05 as determined by ANOVA. (A) GECs were pre-treated with 3-MA for 3 hours prior to *P. gingivalis* infection. (B) GECs were transfected with ATG5 siRNA or non-target siRNA 36 hours prior to *P. gingivalis* infection. Confirmation of ATG5 downregulation (>50%) is shown by Western blot analysis.

There is increasing evidence recently indicating the ability of host-adapted pathogenic bacteria to exploit host autophagic systems for intracellular survival and persistence in the host [42–44]. Our previous studies have explored a variety of host cellular mechanisms *P. gingivalis* employs to successfully survive and replicate in primary GECs [16,20,23,24]. However, the importance of autophagy for optimal survival has not been established in primary GECs. Our data suggests that *P. gingivalis* is housed in ER-rich autophagosomes, therefore, using pharmacological inhibition of autophagy via 3-methyladenine (3-MA) [45,46] and more specific Autophagy protein 5 (ATG5) siRNA (>50% depletion), a critical phagophore forming protein [39], we assessed the effects on *P. gingivalis* intracellular survival using standard antibiotic protection assays [10,11]. The inhibition of autophagy by 3-MA (Fig. 5A) or ATG5 depletion by siRNA (Fig. 5B) significantly decreased the survival of *P. gingivalis* over 24 hours infection, indicating a critical role for autophagy and specifically initial phagophore formation for intracellular *P. gingivalis* survival (*p* < 0.05) .

### P. *gingivalis* contained in autophagic vacuoles are not targeted to lysosomal degradation

**Figure 6.**
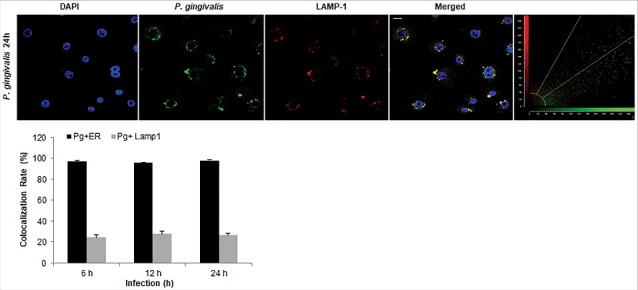
*P. gingivalis'* localization with LAMP1-positive structures in primary GECs. Quantitative confocal microscopic analysis of *P. gingivalis* MOI 100 (Alexa 488) colocalization with lysosomes using the lysosomal marker LAMP1 (Alexa 594) at different time points of infection; 6, 12, and 24 hours. Confocal image represents 24 hours of infection and shows only a small subset of bacteria co-localizing with lysosomes, which remains relatively similar throughout the course of infection. Quantification in the bar graph is shown as the mean +/− SD and graphed with the ER tracker colocalization rates from Figure 4 for comparison. 20x micrographs; Bar 10 µm.

**Figure 7.**
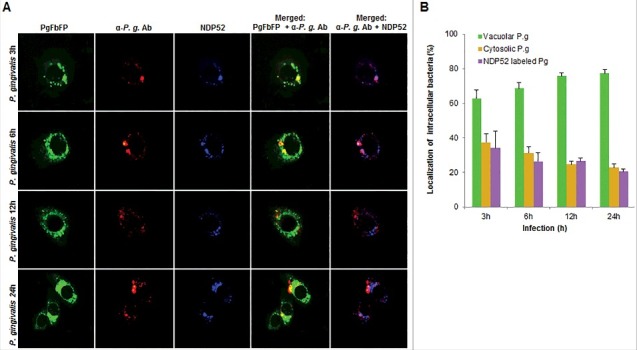
Fluorescence -microscopic analysis of co-localization between vacuolar- and cytosolic-*P. gingivalis* with NDP52 ubiquitin-binding-adaptor protein in primary GECs. (A) Primary GECs were infected with PgFbFP (green fluorescence) and after differential digitonin permeabilization stained with an anti-*P. gingivalis* antibody (Alexa 594, red fluorescence), followed by staining against NDP52 (Alexa 350, blue fluorescence). Cytoplasmic bacteria were detected as FbFP-and Alexa Fluor 594 positive (yellow) previously, whereas vacuolar bacteria were only FbFP-positive (green). 40x micrographs; Bar 10 µm. (B) Majority of cytosolic *P. gingivalis* was determined to show a significant association with NDP52 (red and blue = purple). Mean +/− SD, n>3.

**Figure 8.**
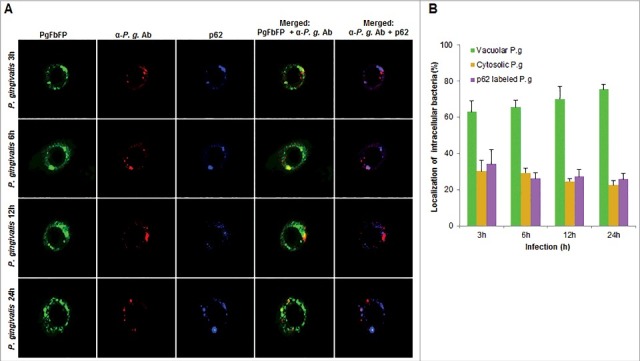
Fluorescence microscopic analysis of co-localization between vacuolar- and cytosolic-*P. gingivalis* with p62 ubiquitin-binding-adaptor protein in primary GECs. (A) Primary GECs were infected with PgFbFP (green fluorescence) and after differential digitonin permeabilization stained with an anti-*P. gingivalis* antibody (Alexa 594, red fluorescence), followed by staining against p62 antibody (Alexa 350, blue fluorescence). Cytoplasmic bacteria were detected as FbFP-and Alexa Fluor 594 positive (yellow) previously, whereas vacuolar bacteria were only FbFP-positive (green). 40x micrographs; Bar 10 µm. (B) Majority of cytosolic *P. gingivalis* was determined to show a significant association with p62 (red and blue = purple). Mean +/− SD, n > 3.

In order to examine whether the bacteria are also associated with lysosomal compartments, primary GECs were infected with *P. gingivalis* and stained with anti-LAMP-1 antibody. The co-localization was quantified and only a small subset of bacteria (∼20%) was shown to be co-localizing with lysosomes (Fig. 6). Since *P. gingivalis* can markedly proliferate in primary GECs, we hypothesized that the small percentage of bacteria co-localized with LAMP-1 are likely the cytosolic bacteria visualized in Fig. 3. Recently, there has been increased interest in the significance of ubiquitin-binding adaptor proteins NDP52 and p62 in the selective targeting of invading bacteria to the lysosomal pathway [47,48]. To determine the possible association of these degradation-targeting intracellular receptors with *P. gingivalis*, primary GECs were infected with PgFbFP strain (green fluorescence) and after employing the selective digitonin permeabilization; the cells were stained with an anti-*P. gingivalis* antibody (red fluorescence), followed by staining against the specific ubiquitin receptor antibodies (blue fluorescence) (Figs. 7 and 8). The fate of autophagic vacuolar (only green) versus cytosolic (red and green = yellow) *P. gingivalis* appeared to be distinctly differentiated by their association with ubiquitin-binding adaptor proteins (Figs. 7 and 8). The quantified results indicate only cytosolic *P. gingivalis* has a significant association (*p* < 0.01) with both the anti-microbial NDP52 and p62 markers (red and blue = purple), which suggests a likelihood of these cytosolic bacteria to be destined to the lysosomal-degradation pathway.

## Discussion

Autophagy is an essential intracellular degradation pathway involved in maintaining cellular homeostasis and is mostly induced in response to a variety of conditions such as Endoplasmic Reticulum (ER) stress, starvation and infections [49]. Through this intracellular process, organelles and cytosolic materials are sequestered into double membrane structures called autophagosomes, which upon maturation, are delivered to a lysosomal compartment for hydrolytic degradation. The role of autophagy in bacterial pathogenesis appear to be multi-dimensional, shown by the characterization of several different autophagy pathways triggered by bacterial invasion leading to either bacterial degradation or enhanced bacterial survival [37]. Several chronic opportunistic pathogens have been demonstrated to evade autophagic-recognition or utilize the autophagy machinery for their own survival [36,42,43]. Some use host autophagic vacuoles for successful replication and persistence (*Salmonella, Mycobacterium, Brucella and Legionella*) while others escape autophagy machinery and thrive in the nutrient rich cytosol (*Shigella* and *Listeria*) [42,50–52].

The ability to propagate and persist in gingival epithelial cells (GECs), which are the first line of defense of oral mucosa, is critical to the success of *P. gingivalis* as an opportunistic pathogen. *P. gingivalis* has been shown to be an important colonizer of the oral cavity, particularly having the skill to intracellularly survive in epithelial cells for extended periods of time [7]. Thus, GECs likely serve as primary reservoir cells for the organism to establish successful colonization in the oral mucosa and disseminate later intercellularly [7,8]. Studies investigating *P. gingivalis*' survival in other cell types suggested variability in the trafficking pattern and fate of the bacterium [34]. For example, in human endothelial cells *P. gingivalis* can be found in autophagosomes [33]; proposed to be within endosomes in KB cells (HeLa) [53]; housed in single membrane vesicles allowing for evasion of bacterial killing in dendritic cells [35]; and inhibition of autophagy increased the survival of *P. gingivalis* in macrophages [54]. These data collectively suggest that the intracellular trafficking and fate of *P. gingivalis* is likely specific to the host cell type. Moreover, essential cellular metabolic events have been shown to be altered both in immortalized and cancer-derived cell lines, therefore accurate results on key metabolic pathways such as molecular trafficking and autophagy can be often hampered by use of the cell lines that already show various de-regulated functions. Thus, there is a need for cellular reductionist models that more closely reflect the *in vivo* infection conditions. At present, *in vitro* primary cells offer invaluable physiologically relevant experimental models to mechanistically study host-pathogen interaction. Nevertheless, the exact mechanisms of *P. gingivalis'* ability to remain unscathed within primary GECs and its intracellular trafficking have not been fully elucidated.

An early study in 1995, using conventional two-dimensional (2D) TEM, showed *P. gingivalis* free in the cytosol during invasion of primary GECs, and hypothesized *P. gingivalis* remained unbound in the cytosol and not encapsulated by endocytic vacuoles [10]. Our study reinforces this former study in showing that analysis of initial *P. gingivalis* invasion in primary GECs demonstrates the bacterial internalization into the host cells is not associated with membrane-bound vacuoles (free in the cytosol). We add to this in highlighting the very early localization of *P. gingivalis* with ER structures specifically. Moreover, in-depth 3D TEM analysis of *P. gingivalis* in primary GECs later infection suggests multiple bacteria housed in double membrane autophagic vacuoles and not freely in the cytosol as previously proposed [10]. Interestingly, the superficial (top) 2D TEM images of those infected cells shown in Fig. 2, **top z16** gave the illusion of cytosolically free bacterium which later revealed to be contained in a double membrane vacuole with other bacteria by the Electron Tomography (3D TEM). Our study also allowed first time quantification and visual detection of cytosolic versus vacuolar *P. gingivalis* in the host cells, which indicated that the bacteria were predominantly found residing in the double-membrane vacuoles. Furthermore, we previously showed that *P. gingivalis* is strongly associated with ER structures and utilizes these ER networks as a prominent subcellular-niche for intracellular location in human primary GECs [40]. Comprehensive analyses of *P. gingivalis* infection in this study revealed as early as 15 minutes, sustained and significant association with ER-networks over the course of 24 hours of infection. The ER structures are characterized as nutritionally rich components and have been shown to be often utilized by persistent microbes to promote successful intracellular replication and survival in the host [55,56]. Moreover, the ER is an important site of initial membrane formation of the phagophore in the autophagy process and autophagosomes can be composed of ER molecules [38,39]. This suggests that *P. gingivalis*, once successfully inside primary GECs, may induce the formation of the phagophore to form its replicative niche. Literature indicates the mechanisms of a few other bacteria that may form their own niche. For example, *Anaplasma phagocytophilum* uses a secreted effector to promote nucleation of the autophagosomes and *Yersinia pseudotuberculosis* form its own compartment which subsequently accumulates markers of autophagy [57,58]. These putative mechanisms for *P. gingivalis* are not entirely explored in this initial study, however the modulation of ROS we previously investigated in *P. gingivalis* infected-GECs [21], could suggest an initial role for ROS in inducing the autophagic vacuole formation early during infection [26,59]. Molecular investigations into this mechanism may provide valuable insight into the bacterial and host components important in forming the replicative niche for *P. gingivalis* and other intracellular bacteria. Similarly, induction of host autophagic flux by *P. gingivalis* infection, which favors microbial survival, may therefore vary depending on the quantity of intracellular bacteria and its secreted effectors present in host cell.

We further reveal *P. gingivalis* induces autophagy in a time-dependent manner and that autophagy induction is critical for *P. gingivalis* intracellular survival in primary GECs. It is important to note that a minor subset of *P. gingivalis* was detected in the cytosol outside of vacuoles. In general, bacteria found in cytosol are prone to be targeted by ubiquitination, a critical step in the autophagy process, mediating the docking and targeting of ubiquitin-coated cargo to autophagosomes for subsequent degradation [60,61]. This has been shown in some studies with *Salmonella Typhimurium* [62], *Shigella flexneri* and *Listeria monocytogenes* [63]. Our examination of the role of the ubiquitin-binding-adaptor proteins NDP52/p62 for targeting of *P. gingivalis* to lysosomal degradation pathway show that *P. gingivalis* successfully evades the anti-microbial ubiquitin-lysosomal compartments when housed in autophagic vacuoles, whereas cytosolic bacteria are likely targeted for degradation.

Finally, *P. gingivalis* modulation of autophagy may also influence other cellular pathways modulated by the infection such as the inflammasome. It is previously reported that *P. gingivalis* inhibits caspase-1 activation and therefore secretion of IL-1β from infected cells [19,23,64,65]. Recent studies propose an overlap between autophagy and inflammasome where autophagy acts as an inflammasome regulator; increased autophagy limits inflammasome activation whereas inhibited autophagy increases caspase-1 activation [66,67]. Intracellular NOD-like receptor inflammasomes may also negatively regulate autophagy creating a two-way communication system between these host signaling pathways [68]. Future studies investigating the cross-talk between these key host pathways could provide valuable information on the cellular networks manipulated by opportunistic pathogens for successful intracellular survival and persistence in the oral cavity.

**Figure 9.**
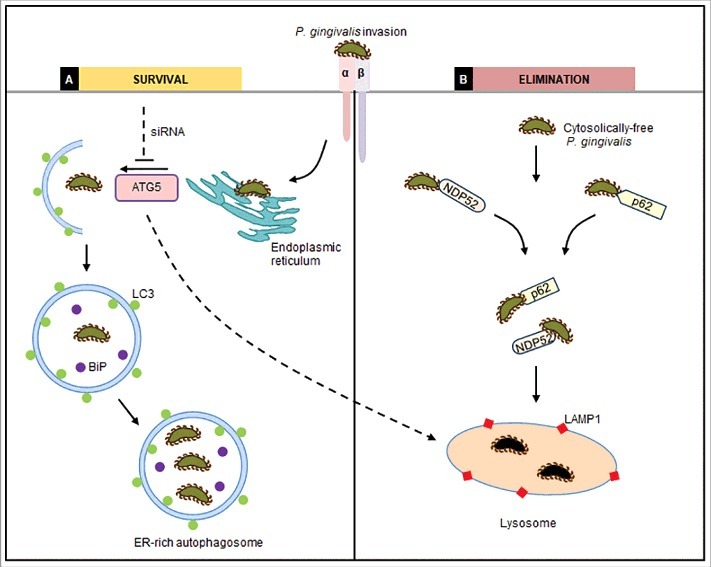
Proposed diagram of *P. gingivalis* trafficking/fate in human gingival epithelial cells. *P. gingivalis* invades primary GECs mainly through specific binding to Beta-1 integrins on the cell surface and the internalized bacteria are not found to be constrained by membrane-bound vacuoles [10–13]. (A) Survival: After initial internalization, cytosolically-free *P. gingivalis* rapidly localizes to ER-rich regions and promotes LC3 lipidation and thus phagophore formation. Multiplying bacteria are found in these ER-rich autophagosomes, marked by LC3 and ER lumen protein BiP, suggestive of a replicative niche in which they are not eliminated. The depletion of ATG5 by siRNA results in significantly less survived intracellular *P. gingivalis* further indicating the importance of autophagy for bacterial life (B) Degradation: Only a small subset of *P. gingivalis* remains free in the cytosol. These cytosolic bacteria are targeted by anti-microbial ubiquitin adaptor proteins, NDP52 and p62, for degradation by the lysosome, also marked by LAMP1.

Overall, our findings indicate a novel mechanism of *P. gingivalis* for affluent survival in primary GECs by exploiting host autophagy. Specifically, in this study we examined the spatio-temporal trafficking of intracellular *P. gingivalis* in GECs and identified ER-rich/LC3 positive autophagic vacuoles which serve as a replicative niche and predominant molecular strategy for the intracellular bacterial survival in the host cells (Fig. 9). Thus, our results indicate *P. gingivalis* utilizes ER-rich-autophagosomes for successful persistence and evades the anti-microbial ubiquitin-lysosomal-degradation pathway. Gaining a better understanding of the molecular mechanisms that contribute to this pro-bacterial autophagy can significantly aid identifying key autophagic target molecules involved in opportunistic bacterial infection, potentially leading to the development of therapeutic intervention strategies aimed at controlling *P. gingivalis'* colonization in the oral cavity.

## Materials and methods

### Bacteria culture

*P. gingivalis* ATCC 33277 was cultured anaerobically at 37°C in trypticase soy broth (TSB) supplemented with yeast extract (1 mg ml^−1^), hemin (5 µg ml^−1^) and menadione (1 µg ml^−1^). *P. gingivalis* was cultured overnight, harvested by centrifugation at 6000 g for 10 minutes at 4 °C, washed twice, and resuspended in Dulbecco's phosphate-buffered saline (PBS) (HyClone). The number of bacteria were quantified using a Klett-Summerson photometer [16]. An inoculum of MOI 100 has been consistently shown to have the optimal attachment and invasion rate in primary GECs and was therefore used throughout this study [8,10,11,16,20,40].

### Primary cell culture

Human primary gingival epithelial cells (GECs) were obtained after oral surgery from adult patients presenting for tooth crown lengthening or impacted third molar extraction. Patients were selected randomly and anonymously and healthy gingival tissue was collected as previously described [16]. The gingival tissue was collected under the approved guidance of the University of Florida Health Science Center Institutional Review Board (IRB, human subjects assurance number FWA 00005790). No patient information was collected and the informed consent was obtained by all subjects. GECs were cultured in serum-free keratinocyte growth medium (KGM, Lonza) at 37°C in a humidified 5% CO_2_ incubator. Primary GECs were used for experiments at ∼75% confluence and were cultured for 24 hours before infection or exposure to other test reagents.

### Transmission electron microscopy

*P. gingivalis* infected cells (15 minutes, 6 hours, and 24 hours) were pelleted by centrifugation and fixed in 4% paraformaldehyde and 1% glutaraldehyde diluted in 1X PBS. Fixed cells were processed with the aid of a Pelco BioWave laboratory microwave (Ted Pella). The samples were washed in 1X PBS and encapsulated in molten 3% low-temperature gelling agarose (Type IV). The encapsulated pellet was water washed and dehydrated in a graded ethanol series: 25%, 50%, 75%, 95%, 100%, 100%, and subsequently infiltrated in HM20 acrylic resin, cured at −20°C with UV light. Cured resin blocks were trimmed, thin sectioned and collected on formvar Ni 400 mesh grids. Sections were post-labeled, post-stained with 2% aqueous Uranyl acetate and Reynold's lead citrate. Sections were examined with a Hitachi H-7000 TEM (Hitachi High Technologies America, Inc.) and digital images acquired with a Veleta camera and iTEM software (Olympus Soft-Imaging Solutions Corp.).

### High-resolution three-dimensional transmission-electron-microscopy

At least 16 consecutive TEM sections were collected from cell samples infected with *P. gingivalis* ATCC 33277 for 12 hours and micrographs of internalized bacterial cells were captured using a Hitachi H-7000 transmission electron microscope (Hitachi High Technologies America, Inc.; the operation voltage is 75 kV) equipped with a Veleta digital camera (Soft Imaging Solutions Corp). The micrographs were converted into aligned mrc image stack files with the Midas program and the newstack command in the IMOD software package (Boulder Laboratory of 3D Electron Microscopy of the Cell, University of Colorado at Boulder). 3D models were generated from the image stacks with the 3dmod program (IMOD software package).

### Immunofluorescence using flavin mononucleotide (FMN)-green-fluorescent transformant-strain (PgFbFP)

Primary GECs were seeded at a density of 7 × 10^4^ on glass coverslips in four-well plates. Cells were infected with PgFbFP, constructed as previously described [40], at an MOI of 100 and incubated for 24 hours. Cells were then immediately washed three times with buffer (100 mM KCl, 20 mM HEPES, 2 mM MgCl_2_) and treated with the selective permeabilizing agent, digitonin (50 ug ml^−1^) (Sigma), for 2 minutes at room temperature [41]. The cells were then incubated at 37°C with rabbit anti-*P. gingivalis* ATCC 33277 antibody (1:1000) for 20 minutes to label cytosolic bacteria, followed by staining with anti-rabbit Alexa Fluor 594 conjugated secondary antibody 1:1000 (Invitrogen) for 20 minutes at 37°C. The immunostained cells were mounted on glass slides using Vectashield mounting media with DAPI, and examined using wide-field fluorescence microscope (Zeiss Axio Imager A1). The images were captured using a cooled charge-coupled device camera controlled by QCAPTURE software (Qimaging). Cytosolic bacteria were detected as Alexa Fluor 594 positive while vacuolar bacteria were only FbFP positive. Immunofluorescence intensity based quantification of cytosolic and vacuolar *P. gingivalis* at different time points was carried out using NIH Image J analysis. To detect NDP52 and p62, the same staining protocol was followed using these specific antibodies: rabbit anti-NDP52 1:50 (Abcam, ab68588) and mouse anti-p62 1:50 (BD Biosciences, 610833) followed by staining with Alex Fluor 350 conjugated secondary antibody 1:1000 (Invitrogen). The immunostained cells were mounted on glass slides using Vectashield mounting media without DAPI.

### LC3 western blot analysis

Primary GECs seeded at a density of 2 × 10^5^ on six-well plates, were infected with *P. gingivalis* ATCC 33277 at an MOI of 100 for 0.5, 1, 3, 6, 12, and 24 hours. Cells were lysed with1X RIPA lysis buffer (Cell Signaling) with protease and phosphatase inhibitors: 1mM PMSF; 0.1mM TLCK; 1mM NaF; 2mM N-ethylmaleimide; 1 mM sodium orthovanadate; and 10 µg ml^−1^ aprotinin. Equal amounts of total protein were determined by colorimetric Bradford Assay (Bio-Rad) and loaded onto a 15% polyacrylamide gel. After gel electrophoresis, the proteins were transferred onto a nitrocellulose membrane using wet-transfer system and the membrane was blocked in Tris-buffered saline with 0.1% Tween 20 (TBST) containing 5% nonfat dry milk.

Polyclonal rabbit anti-LC3 antibody was used at a dilution of 1:1000 (Novus Biologicals) and detected using anti-rabbit HRP-conjugated secondary antibody at 1:1000 (Cell Signaling). The same membrane was further stripped and probed with mouse anti-ß-tubulin antibody antibody 1:1000 (Invitrogen) for loading control, followed by anti-mouse HRP-conjugated secondary antibody 1:1000 (Cell Signaling). Protein bands were visualized using enhanced chemiluminescence (ECL, GE Healthcare) and band intensities were examined using NIH ImageJ.

### GFP-LC3 construct and transfection

cDNA encoding human LC3 was obtained by RT–PCR from human primary GECs total cDNA with the LC3-forward primer (5′-AAGCTTATGCCGTCGGAGAAGA-3′) and LC3 reverse primer (5′-GAATTCTTACACTGACAATTTCATCCC-3′). It was then subcloned into the pGEM-T vector (Promega). To construct pEGFP-LC3 plasmid, LC3 cDNA was inserted into the HindIII and EcoRI sites of pEGFP-N1, a GFP fusion protein expression vector (Clontech Laboratories). The completed GFP-LC3 plasmid was verified by DNA sequencing. Human primary GECs were plated at a density of 7 × 10^4^ on glass coverslips in four-well plates and cells were stably transfected with GFP-LC3 plasmid DNA for 36 hours using Glycofect (KeraFAST) in serum free KGM medium (Lonza) following the manufacturer`s instructions.

### Confocal microscopy

Human primary GECs were plated at a density of 7 × 10^4^ on glass coverslips in four-well plates and cultured until ∼70% confluence. Cells were infected with *P. gingivalis* ATCC 33277 at a multiplicity of infection (MOI) of 100 and incubated for 3, 6, 12, and 24 hour time periods. The infected live cells were incubated with ER-Tracker Red (Invitrogen) following the manufacturer's instructions. Cells were fixed with 10% Neutral buffered formalin, permeabilized by 0.1% Triton X-100, and stained for 1 hour at room temperature with anti-*P. gingivalis* ATCC 33277 rabbit polyclonal antibody (1:1000). The stained cells were washed and incubated for 1 hour at room temperature with anti-rabbit Alexa Fluor 488 conjugated secondary antibody (1:1000) (Invitrogen). The immunostained cells were mounted on glass slides using Vectashield mounting media with DAPI. Co-localization of *P. gingivalis* with regards to ER in different time points of post infection was observed by confocal microscopy and quantified using co-localization analysis tool in the LAS AF software (LeicaTCS-SP5 Confocal). Analysis of LAMP-1 was conducted using mouse anti-LAMP1 1:500 (Abcam, ab25630) followed by anti-mouse Alexa Fluor 594 (Invitrogen).

Cells were transfected with GFP-LC3 plasmid DNA for 36 hours and then infected with *P. gingivalis* ATCC 33277 at an MOI of 100. Cells were then fixed with 10% neutral buffered formalin for 30 minutes at room temperature and permeabilized for 20 minutes with 0.01% Triton X-100 and blocked for 30 minutes with PBS containing 3% BSA. For immunofluorescence staining, cultures were stained for 1 hour at room temperature with anti-*P. gingivalis* ATCC 33277 rabbit polyclonal antibody. The stained cells were washed and incubated for 1 hour at room temperature with Alexa Fluor 594 secondary antibody 1:1000 (Invitrogen). Punctated GFP-LC3 during *P.gingivalis* infection was examined by confocal microscopy (LeicaTCS-SP5 Confocal). Co-localization of *P. gingivalis* with GFP fluorescence was quantified using the JACoP tool under NIH ImageJ software.

### Immunofluorescence

Primary GECs were seeded at a density of 7 × 10^4^ on four-well plates, which were infected with *P. gingivalis* ATCC 33277 at an MOI of 100 for 24 hours. Cells were then incubated with blocking buffer (PBS, 0.1% Triton-X, 2% BSA) for 20 minutes, followed by incubation of primary antibodies of rabbit anti-*P. gingivalis* ATCC 33277 1:1000, mouse anti-LC3 1:1000 (Cell Signaling) or goat anti-Bip (1:1000) (Santa Cruz). Stained cells were washed once and incubated with the respective conjugated secondary antibodies, Alexa Fluor 488, Alexa Fluor 594, or Alex Fluor 350 1:1000 (Invitrogen), respectively. Both primary and secondary antibodies were incubated for 1 hour at room temperature. The immunostained cells were mounted on glass slides using Vectashield mounting media with or without DAPI, and examined using wide-field fluorescence microscope (Zeiss Axio Imager A1). The images were captured using a cooled charge-coupled device camera controlled by QCAPTURE software (Qimaging).

### siRNA ATG5 transfection and western blot

Western blot analysis was performed to confirm ATG5 knockdown. Primary GECs at 80% confluence were transfected in serum free KGM medium (Lonza) with 5 pmol of pre-designed ATG5 siRNA duplexes (siRNA ID#: s18160; Ambion) using lipofectamine RNAiMAX Reagent (Invitrogen) for 48 hours or Non-target pool siRNA (Dharmacon). Colorimetric Bradford Assay (Bio-Rad) was used to determine protein concentrations of the transfected and samples. Equal amount of protein samples were subjected to 12% sodium dodecyl sulfate-polyacrylamide gel electrophoresis (SDS-PAGE). After gel electrophoresis, the proteins were transferred onto a nitrocellulose membrane using wet-transfer system and the membrane was blocked in Tris-buffered saline with 0.1% Tween 20 (TBST) containing 5% nonfat dry milk. The membrane was then incubated with rabbit anti-ATG5 antibody at a dilution of 1:500 (Cell Signaling) and treated with anti-rabbit HRP-conjugated secondary antibody at 1:1000 (Cell Signaling). The blot was then stripped and probed with mouse anti-ß-tubulin antibody 1:1000 and anti-mouse HRP-conjugated secondary antibody (Cell Signaling) at 1:1000. Protein bands were visualized using enhanced chemiluminescence (ECL, GE Healthcare) and band intensities were examined using NIH ImageJ.

### Antibiotic protection assays

Primary GECs were seeded at a density of 2 × 10^5^ on six-well plates and were treated with an autophagy inhibitor, 3-methyladenine (3-MA) (Sigma), 3 hours prior to *P. gingivalis* infection. The cells were then infected with *P. gingivalis* at an MOI of 100 for 3, 6, 12 and 24 hours with or without the inhibitor. To quantify the intracellular viability of *P. gingivalis* within GECs treated with or without the inhibitor, antibiotic protection assay was performed. At each time of collection, cells were washed three times with PBS and incubated for 1 hour in KBM with metronidazole (200 μg ml−1) and gentamicin (300 μg ml−1). Cell supernatants were sampled as control to confirm the extracellular bacterial killing. Cells were then lysed with 1% Triton X-100 and the number of colony forming units (CFU) was enumerated by serial dilution of lysates in PBS and plating on sheep blood agar plate supplemented with hemin (5 µg ml−1) and menadione (1 µg ml−1). For ATG5 experiments, primary GECs were transfected in serum free KGM medium (Lonza) with 5 pmol of pre-designed ATG5 siRNA duplexes (siRNA ID#: s18160; Ambion) using lipofectamine RNAiMAX Reagent (Invitrogen) for 36 hours then infected with *P. gingivalis* ATCC 33277 at an MOI of 100 for 3, 6, 12, 24 hours. Transfection was performed following the manufacturer's instructions. Non-target pool siRNA (Dharmacon) and *P. gingivalis* infection alone were used as negative controls.

### Statistical analysis

Either one-way ANOVA or two-tailed Student's t-test were used to evaluate significance. P-values of 0.05 or less were considered to be statistically significant. All experiments were performed at least 3 separate occasions with an n≥3.

## Supplementary Material

Supplementary_Figure_1B_Volocity_Video.avi

## Abbreviations and acronyms

CFU: colony forming units
GEC: gingival epithelial cell
MOI: multiplicity of infection
